# Anisotropic Thermal Properties of Electrically Insulating Thin Films and Sheets: A Review

**DOI:** 10.1002/smtd.71012

**Published:** 2026-09-24

**Authors:** Shanmukhi Sripada, Cole R. Davis, Aalok U. Gaitonde, Amy M. Marconnet

**Affiliations:** ^1^ Birck Nanotechnology Center and The School of Mechanical Engineering Purdue University West Lafayette Indiana USA; ^2^ Naval Surface Warfare Center Crane Division Crane Indiana USA

**Keywords:** anisotropy, composites, nanomaterials, nanotubes, thermal conductivity, thermal transport

## Abstract

With the growing need for controlling heat dissipation in modern technologies, a comprehensive understanding of thermal transport in electrically insulating materials is essential. Electrically insulating thin films and sheets often exhibit anisotropic thermal conductivity across in‐plane and cross‐plane directions, either intrinsically, or through structural engineering. This anisotropy enables targeted heat dissipation, such as around sensitive components in modern electronics. This review summarizes the recent progress in enhancing the thermal conductivity of electrically insulating thin films and sheets, in particular, those exhibiting anisotropy in the thermal conductivity. It considers how fabrication strategies impact the structure of the material to control thermal conductivity and thermal conductivity anisotropy, and summarizes the techniques leveraged to measure the thermal conductivity, and evaluates the corresponding electrical parameters. Trends across various material systems, including polymers, ceramics, and composites, are discussed, with additional insights into thickness‐dependent behavior, compositing strategies, alignment methods, and trends in filler loadings. Special attention is given to boron nitride (BN)‐based composites, where filler alignment, morphology, and loading fraction critically influence anisotropic thermal performance. By analyzing these interrelationships, this review provides a framework for designing next‐generation insulating materials with tunable anisotropic thermal properties for advanced thermal management applications.

## Graphical abstract

Thermally conducting and electrically insulating materials with anisotropy properties play an integral role in advanced thermal management, in particular for electronic devices. This review discusses recent progress in enhancing thermal conductivity and controlling the thermal anisotropy between in‐plane and cross‐plane (through‐thickness) directions. By analyzing the interrelationships, this review provides a framework for designing next‐generation insulating materials with tunable anisotropic thermal properties for advanced thermal management applications.

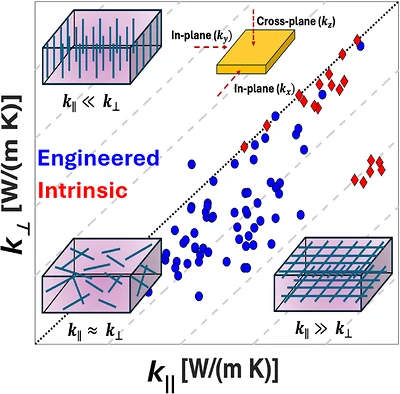

## 1 Introduction

Heat generated in electronic and electrical devices must be dissipated to ensure reliability and operation below temperature limits. To properly manage this thermal energy, it must flow through different materials in a series of thermal management components that may include thermal interface materials (TIMs), heat spreaders, and heat sinks [1, 2]. Thermal design encompasses creating and optimizing systems or components to manage heat transfer efficiently and maintain operation within safe temperature thresholds. This process includes understanding the mechanisms of heat transfer within the system, designing and selecting materials with appropriate thermal properties for different components, and developing optimal active and passive cooling solutions. Materials selection becomes important for thermal management applications and often requires a selection of materials with a unique combination of thermal and electrical properties.

Electrically insulating materials play a critical role in modern technologies, including microelectronics, flexible devices, power modules, electric motors, aerospace systems, and energy storage platforms [3, 4, 5]. Beyond their fundamental function of preventing charge leakage and short circuiting, these materials are critical links in the heat transfer pathways and must accommodate increasingly severe thermal loads as device power densities rise. Effective heat dissipation is critical for ensuring operational stability, extending device lifetime, and enabling further miniaturization. However, the dual requirement of electrical insulation and efficient thermal management presents a persistent challenge: most conventional electrical insulators, such as polymers, exhibit inherently low thermal conductivity (< 0.3 W $m^{-1}$ $K^{-1}$ [6]) and thus, poor thermal management capabilities. Therefore, extensive research has been conducted to improve the thermal performance of polymers and their composites, while maintaining the electrical performance to enhance their suitability for such demanding applications.

In addition to the ability to move heat effectively (thermal conductivity), the anisotropy in thermal conductivity (i.e., the ratio between in‐plane and cross‐plane thermal conductivity) offers another valuable design parameter to design materials with enhanced thermal performance, as the heat can be directed or corralled in a system passively. Traditional isotropic materials distribute heat uniformly in all directions, which can lead to inefficient thermal routing and parasitic thermal cross‐talk between adjacent components in densely packed architectures. Materials with high in‐plane conductivity can act as efficient lateral heat spreaders, while low cross‐plane conductivity may serve to insulate and confine heat or reduce thermal leakage across interfaces (protecting delicate or temperature‐sensitive components). This is particularly relevant in applications such as microelectronics, power devices, and flexible electronics. In applications like electric motor windings, it would be useful to have high cross‐plane thermal conductivity to enhance dissipation away from the surrounding copper windings. In layered systems, films, and composites, anisotropy can thus be deliberately exploited to achieve tailored thermal pathways. Such control is particularly relevant in applications including TIMs, insulating coatings, and flexible substrates, where direction‐dependent thermal transport directly impacts device reliability and performance.

Anisotropy in electrically insulating materials arises from a range of intrinsic and engineered mechanisms. Crystalline symmetry, preferred orientation, and structural layering impart strong anisotropic behavior in intrinsic materials, while processing methods such as extrusion, ice‐templating, or alignment of high‐conductivity fillers can create anisotropy in polymers and composites. For thin films and sheets (where thickness is much smaller compared to the lateral dimensions), where directional confinement is already intrinsic, fabrication strategies become especially critical in tuning the in‐plane and cross‐plane thermal transport balance.

A few recent reviews have focused on thermally conductive, electrically insulating polymer composites and related materials, reflecting the increasing demand for advanced thermal management in electronic, energy, and structural applications. Lin et al. [7] briefly reviewed isotropic polymer nanocomposites with high in‐plane and cross‐plane thermal conductivity. Their primary focus was on diverse applications and the critical need to balance thermal conductivity with other application‐specific material properties. Khan et al. [8] briefly reviewed isotropic nitride‐based polymer composites (BN, AlN, Si_3_N_4_, C_3_N_4_, TiN) and their role in boosting mechanical, thermal, and dielectric properties. Singh et al. [9] provided a detailed review on polymer composites, particularly containing Boron Nitride nanofillers, discussing challenges in filler quality, orientation, and measurement calibration, while noting the complications of tailoring in‐plane versus cross‐plane thermal conductivities. Other reviews have explored related topics: BN nanosheets integrated in composites designed for construction applications [10]; preparation strategies and filler morphologies (focusing on filler‐matrix interactions and industrial challenges) for thermally conductive polymer composites [11, 12, 13]; three‐dimensional (3D) thermally conductive boron nitride (BN)/polymer composites [14]; enhancing the out‐of‐plane thermal conductivity in BN/polymer composites [15]; core–shell fillers as a scalable solution for maintaining insulation with high thermal conductivity [16]; and electrically insulating composites for electrical machines and ceramic/polymer systems with aligned fillers with focus on cross‐plane thermal conductivity [17].

Taken together, these reviews demonstrate the broad interest in thermally conductive, electrically insulating composites, particularly polymer‐ and BN‐based systems. However, the existing literature has several key limitations. First, most reviews emphasize isotropic thermal transport or focus separately on in‐plane or cross‐plane enhancements, rather than analyzing anisotropy systematically. Second, few have addressed thin films and sheets, where confinement effects and thickness scaling critically influence anisotropy ratios and thermal conductivities. Third, while BN composites are often discussed, their anisotropy ratio versus filler loading, orientation, form factor, and processing has not been comprehensively summarized. Fourth, most of the reviews focus on a single category of materials or filler particles, rather than considering a broad range of materials. Finally, most reviews typically target thermal properties without considering other important factors. For practical applications, electrical properties like dielectric breakdown strength, dielectric constant, and volume resistivity play an equally important role.

This review directly addresses these gaps by providing a systematic, data‐driven analysis of anisotropic thermal properties (focusing on both in‐plane and cross‐plane thermal conductivity) across a diverse spectrum of electrically insulating thin films and sheets. Rather than limiting the scope to boron nitride (BN)‐based systems, this work comprehensively evaluates multiple material classes, including intrinsically anisotropic materials and engineered materials including pure polymer films and fibers, and composites with different types of electrically insulating fillers including BN and AlN. In addition, this review extends beyond thermal properties to evaluate the essential dielectric parameters required for practical engineering applications. Furthermore, this review simultaneously reviews the most common thermal conductivity measurement techniques that have been used for characterizing such materials. Section 2 surveys the different classes of thermally anisotropic materials (i.e., those with intrinsic versus engineered anisotropic thermal conductivity) and the fabrication strategies used to induce or control anisotropy. Section 3 briefly summarizes measurement techniques for characterizing the thermal conductivity of thin films and sheets. Section 4 analyzes trends in thermal conductivity and anisotropy ratio across insulating material classes, including polymers, composites, and pure intrinsic materials, and additionally highlights thickness‐dependent behaviors. Special attention is given to boron nitride (BN)‐based composites, where filler alignment, form factor, and loading fraction strongly dictate anisotropic performance. Section 5 evaluates the corresponding trends in key electrical properties, including dielectric breakdown strength, dielectric constant, and volume resistivity. By mapping out these relationships, this review aims to provide a framework for designing next generation insulating materials with tailored anisotropic thermal properties for advanced thermal management applications.

## 2 Anisotropic Materials and Fabrication Methods

This review focuses on electrically insulating materials exhibiting anisotropic thermal conductivity. These materials can be further divided into two categories: intrinsic materials and engineered materials. *Intrinsic materials* are defined here as materials having intrinsic or natural anisotropic thermal conductivity because of their inherent crystal structure and/or atomic bonding. *Engineered materials* are defined here as materials where the anisotropy in thermal conductivity or improvements in anisotropy are introduced due to fabrication method or intentional materials design. Engineered materials may utilize a combination of intrinsic materials and fabrication techniques to achieve optimal anisotropy. The following sub‐sections delve into the details of these two classes of materials.

### 2.1 Intrinsic Materials

Few electrically insulating materials display intrinsic anisotropic thermal conductivity in their bulk form (i.e., as macroscopic, standalone solids). These materials are generally ceramics including boron nitride (BN), silicon nitride (Si_3_N_4_), aluminum nitride (AlN), and sapphire (Al_2_O_3_). Table 1 summarizes their key characteristics, including crystal structures, thermal conductivity parallel ($k_{∥}$) and perpendicular ($k_{⊥}$) to the basal plane/c‐axis, dielectric breakdown strength ($E_{b}$), and dielectric constant ($\varepsilon^{\prime }$).

**Table 1:** Typical characteristics, thermal properties, and electrical properties of selected intrinsically anisotropic, electrically insulating bulk materials.

Material	Structures	Thermal properties	Electrical properties	Other characteristics	Refs.
BN	h‐BN and pyrolytic BN	$k_{∥}∼$300‐600 W/(m K) $k_{⊥}∼$2–5 W/(m K) AR∼85–170	$E_{b}∼$400–1000 kV/mm $\varepsilon^{\prime }∼$3–4	Extreme Thermal and Chemical Stability, High Resistance to Oxidation	[9, 22]
Si_3_N_4_	α‐Si_3_N_4_ and β‐Si_3_N_4_	$k_{∥}∼$70–180 W/(m K) $k_{⊥}$ ∼25–90 W/(m K) AR∼1.2–3	$E_{b}∼$35.4–47 kV/mm $\varepsilon^{\prime }∼$2.5–8	High Resistance to Oxidation, Creep, and Fatigue, Good Fracture Toughness	[8, 23, 24]
AlN	Wurtzite	$k_{∥}∼$40–300 W/(m K) $k_{⊥}∼$40–300 W/(m K) AR∼1–1.1	$E_{b}∼$50–150 kV/mm $\varepsilon^{\prime }∼$8.6–9	Low CTE, Low Cost, Good Wear and Tear Resistance	[8, 25, 26]
Al_2_O_3_	Rhombohedral	$k_{∥}∼$7–50 W/(m K) $k_{⊥}∼$7–35 W/(m K) AR∼1–1.5	$E_{b}∼$50 kV/mm $\varepsilon^{\prime }∼$8.6–9.3	High Hardness, High Chemical Inertness, High Resistance to Oxidation, High Rensile Strength	[27, 28]

Boron nitride (BN) exists in several polymorphs with the two most common being hexagonal and cubic. Of these, only hexagonal boron nitride (*h‐*BN) exhibits anisotropic thermal conductivity due to its layered structure similar to graphite [18]. A form of *h*‐BN fabricated by chemical vapor deposition (CVD) is referred to as pyrolytic boron nitride (PBN) and also has anisotropic thermal conductivity [19]. In *h*‐BN, alternating bonds of boron and nitrogen are arranged in a hexagonal structure to form layers or sheets that are stacked in an “ABAB” sequence. Weak Van der Waals forces hold the sheets together and contribute to the material's anisotropy by reducing phonon transport between sheets while phonon transport is high along the covalently bonded crystalline planes [20, 21].

Silicon nitride (Si_3_N_4_) is commonly found in two polymorphs, alpha (α) and beta (β). Both polymorphs have a hexagonal crystal structure but differ in their stacking sequence, where α‐Si_3_N_4_ is “ABCD” and β‐Si_3_N_4_ is “ABAB”. Both polymorphs display anisotropic thermal conductivity; however, β‐Si_3_N_4_ is more commonly associated with this due to its elongated, rod‐like grains that accentuate this anisotropy [29, 30]. The β‐Si_3_N_4_ grains themselves exhibit higher thermal conductivity along the *c*‐axis (i.e., along the length of the rod) due to the bonding structure and low defect density [30]. Not only does the crystal structure of bulk Si_3_N_4_ contribute to the anisotropic thermal conductivity, but the grain size and orientation are also important factors [29, 31]. For example, tape casting can orient β‐Si_3_N_4_ to enhance the anisotropic behavior through microstructure control [30, 32, 33]. While grains provide good phonon transport, grain boundaries scatter phonons and reduce thermal conductivity. When grains are elongated and aligned, there are fewer scattering events (i.e., grain boundaries) along the alignment direction and thus, a larger thermal conductivity. Hot pressing can also be used to oriented β‐Si_3_N_4_, while extended sintering times and β‐ Si_3_N_4_ seed particles can be used to promote conversion of α‐Si_3_N_4_ to β‐Si_3_N_4_ and further improve anisotropy [29, 31, 32, 33, 34, 35].

Aluminum nitride (AlN) in its wurtzite crystal structure exhibits weak anisotropic thermal conductivity. This hexagonal lattice, characterized by alternating Al and N layers, yields only a 4%–6% difference between the c‐axis and a‐axis, as predicted by first‐principles calculations [36, 37, 38]. Unlike strongly anisotropic layered materials such as graphite or *h*‐BN, wurtzite AlN is not Van der Waals bonded; instead, comparable covalent/ionic bonding along both c and basal directions results in similar phonon velocities and elastic constants, and hence only minor anisotropy in heat transport. Despite this weak anisotropy, AlN is technologically important due to its exceptionally high thermal conductivity (∼300 W $m^{-1}$ $K^{-1}$ in films < 10 µm thick), making it one of the promising candidates for heat‐spreading applications in microelectronics [39].

Aluminum oxide (Al_2_O_3_) also displays relatively weak (10%–20%) intrinsic anisotropic thermal conductivity. Specifically, sapphire, a rhombohedral single crystal of Al_2_O_3_, is most cited for having anisotropy with the largest thermal conductivity being observed along the *c*‐axis of the crystal [40, 41].

### 2.2 Engineered Materials

In this review, we use the term “engineered materials” to describe materials that exhibit anisotropy (in thermal conductivity) through material design including creation of composites and utilization of materials with intrinsic anisotropic thermal conductivity. Specifically, we evaluate polymer films and fibers (Sub‐Section 2.2.1) and composites (Sub‐Section 2.2.2).

#### 2.2.1 Polymer Films and Fibers

Bulk polymers typically exhibit isotropic thermal conductivity, but chain alignment during fabrication or conversion into fibers can induce anisotropy, especially for thin films, sheets, and fibers. This effect has been demonstrated in materials such as meta‐aramids, polyesters, cellulose, and polyimides, and is widely exploited in commercial electrical insulation materials like fish paper, Nomex, and Kapton. For example, Nomex 410 [42] and fish paper [43] are produced by wet‐lay papermaking, where short fibers (aramid and cellulose produced by wet spinning and chemical pulping of wood, respectively) are dispersed in a slurry, formed into sheets, then pressed and dried. This process aligns fibers in the sheet plane, yielding enhanced in‐plane thermal conductivity [44]. In contrast, Kapton films are made by casting a polyamic acid precursor and thermally imidizing it into a continuous polyimide film [45], resulting in a more uniform structure, though molecular orientation during curing can still introduce anisotropy.

In multilayer laminates such as Nomex‐based laminates (such as NMN consisting of Nomex‐Mylar‐Nomex) or 3M^TM^ TufQUIN Triplex materials, different sheets (e.g., Nomex and Mylar) are bonded with resins and calendered (i.e., passed through heated rollers under pressure) to achieve uniform thickness, smooth surfaces, and strong adhesion between layers. These interfaces further accentuate anisotropy, particularly through the thickness, due to material discontinuities. Overall, anisotropy arises from sheet‐forming processes in which reinforcing elements preferentially align in‐plane, enhancing in‐plane transport.

#### 2.2.2 Composites

Integrating filler particles into bulk polymers or ceramics is one of the most common methods to tailor the thermal conductivity of a bulk material, as it allows for precise control over the material composition and microstructure. Furthermore, the anisotropy in thermal conductivity can be controlled by aligning or orienting the filler materials, especially when integrating high aspect ratio fillers (such as fibers). While polymer matrix composites (PMCs) are common (likely due to the manufacturability and durability of PMCs), ceramic matrix composites (CMCs) are also of interest as electrically insulating materials, especially in high‐power systems.

Common filler materials for electrically insulating, thermally conducting composites are BN and AlN. The bulk forms of both materials (see Section 2.1) intrinsically demonstrate anisotropic thermal conductivity. Boron nitride fillers come in a variety of forms, including nanotubes (BNNTs), nanosheets (BNNSs) or platelets, and *h*‐BN particles. In contrast, AlN fillers are more commonly whiskers, powders, and flakes. While both fillers exhibit intrinsic anisotropic thermal conductivity, composites including these materials are categorized herein as *engineered materials* and not intrinsic materials due to the need to create composites or architected materials to utilize their anisotropy at the macroscale. For example, for electrical insulation applications, BNNTs are not typically used as individual, isolated nanotubes, but commonly they are engineered into non‐woven mats or buckypapers, creating bulk fabrics that can be integrated into systems [46, 47, 48]. To fabricate these materials, the nanomaterials are dispersed in a solvent followed by vacuum‐assisted filtration. Proper dispersion prevents aggregation so that the material can self‐assemble and align along the filtration media surface, creating a fibrous, fabric‐like material. These materials often exhibit good alignment and anisotropy due in part to the orientation of the nanofillers and their intrinsic anisotropy [46]. Buckypaper‐type materials can also be fabricated with two or more nanomaterials, like BNNTs and cellulose nanofibers (CNF) [49], to create a composite fabric. Buckypapers are naturally porous, which makes them suitable candidates for infiltration with a matrix to form engineered composites.

Various fabrication methods have been developed to produce composites (including those with BN and AlN fillers) with anisotropic thermal conductivity. The alignment of fillers critically influences the directional thermal conductivity. As illustrated in Figure 1, different alignment strategies lead to distinct thermal transport behaviors: randomly oriented fillers result in nearly isotropic conductivity (Figure 1(b)); cross‐plane alignment enhances cross‐plane conductivity (Figure 1(c)); in‐plane alignment increases in‐plane conductivity (Figure 1(d)); and unidirectional in‐plane alignment produces pronounced in‐plane anisotropy, with thermal conductivity along the filler direction exceeding both the other in‐plane and cross‐plane values (Figure 1(e)).

**Figure 1:**
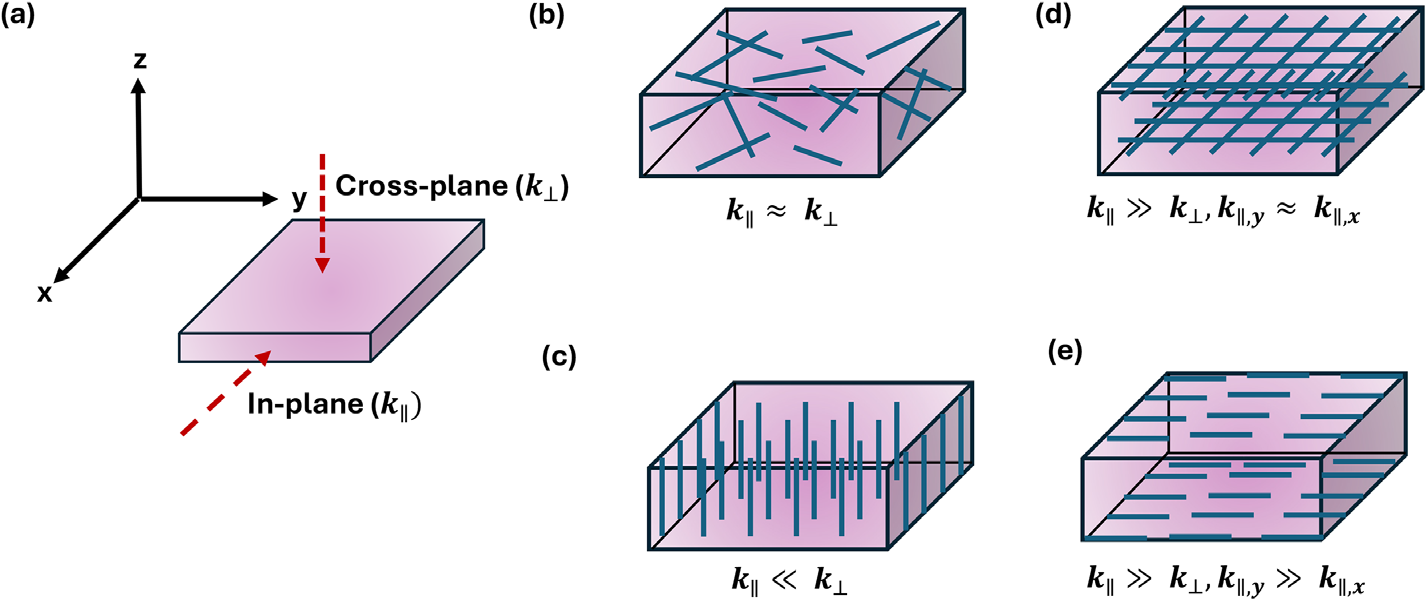
Schematic of thermal conductivity measurement directions and filler alignment in composites. (a) In‐plane (∥) and cross‐plane (⊥) measurement orientations. (b) Randomly oriented fillers, yielding nearly isotropic thermal conductivity. (c) Through‐thickness alignment, enhancing cross‐plane thermal conductivity. (d) In‐plane alignment, increasing in‐plane thermal conductivity. (e) Unidirectional in‐plane alignment, producing pronounced in‐plane anisotropy with higher thermal conductivity along the filler direction relative to the other in‐plane and the cross‐plane directions.

For composite materials, alignment and anisotropy is most commonly achieved through an applied force (e.g., shear), as opposed to other methods like magnetic and electrical fields. Although alignment of BN platelets via magnetic field has been achieved, modification using magnetic particles (e.g., iron oxide) was required to create magnetically responsive BN platelets [50, 51]. This method is excluded from this review as the addition of metallic particles diminishes electrically insulating properties. Similarly, electrical fields have been used to align BNNSs, but require semiconductor additives like titanium dioxide [52] or are limited by filler loading [53]. Therefore, physical methods of alignment like hot pressing and fiber drawing (e.g., electrospinning) are typically used to maintain electrical insulation.

Hot pressing is a common method to fabricate composites with aligned fillers resulting in anisotropic thermal conductivity. This method is combined with a variety of dispersion and mixing methods prior to hot pressing including melt, solution [54, 55, 56], and latex [57] blending, ball milling [58, 59, 60], bidirectional freezing [11], spray‐coating [56], surface modification and deprotonation [61], and melt compounding with fibrillation [62]. Hot pressing relies on shear forces to align the filler materials perpendicular to the pressing direction. However, some dispersion techniques like melt compounding with fibrillation and bidirectional freezing provide alignment on their own, and hot pressing is used to densify the material afterward. But, hot pressing after filler alignment during a process like fibrillation has been observed to reduced alignment and anisotropy [62]. While hot pressing does not always result in maximum anisotropy, it does provide alignment in a relatively simple fabrication technique.

Another alignment technique is to fabricate fibers or nanocomposite fibers. Continuous *h*‐BN fibers (∼10 µm diameter) have been synthesized from a polyborazine preceramic polymer using melt extrusion. While the specific anisotropic properties were not measured, this material shows promise as a thermal management material [63]. Composite fibers from PMIA and BNNS have also been fabricated and show a cross‐plane and in‐plane thermal conductivity of 0.833 and 5.6 W $m^{-1}$ $K^{-1}$, respectively [64]. Recent studies by Smith et al. [65, 66, 67] developed a method to fabricate BNNT fibers from wet spinning with PAN. Although their thermal conductivity was not measured, the resulting fibers had low void content and high tensile strength giving promise for high thermal conductivity. Nanocomposite fibers are more commonly cited in literature and are often fabricated via electrospinning. Briefly, electrospinning is a process used to extrude fibers from a polymer solution. When combined with nano material fillers, this technique is used to fabricate polymer‐based nanocomposite fibers. This process first requires the dispersion of the BN‐based filler in a polymer solution sometimes requiring sonication [68, 69] or even surface modification [70] to achieve a good dispersion. This solution is then extruded from a syringe tip and wound onto a spinning drum. Shear forces from the nozzle extrusion and elongation from fiber winding provide alignment of the filler material along the fiber direction [69, 70]. These BN‐loaded polymer fibers can be used as‐is but contain significant porosity (>50%) [68]. To eliminate porosity, post processing is required by stacking fiber sheets and hot pressing [70, 71]. Benefits to this method include controlled alignment of the filler to promote anisotropy and even control of the in‐plane alignment. However, some drawbacks may include producing low‐density materials that require post‐processing, difficulties in achieving good dispersions, and limits to filler loading (<33 wt.% [71]).

While hot pressing and fiber drawing or electrospinning are common methods to achieve alignment and anisotropy, several other methods have been explored including bi‐directional freezing [72], ice templating [73, 74], and vacuum filtration [75, 76] followed by matrix infiltration, ice pressing [77], digital light projection 3D printing [78], high shear roll milling [79], injection molding [80], melamine‐formaldehyde sponge coating and pressing [81], blade casting [82], solution casting [83], and spin casting [84].

## 3 Thermal Conductivity Measurement Techniques

In order to understand the thermal properties of these types of materials, several different techniques have been developed. Both the in‐plane and cross‐plane thermal conductivity are important for thermally thin heat spreading materials (with form factors of thin films or sheets). Further, the in‐plane thermal conductivity can depend on direction. Thermal conductivity measurements involve heating a sample to create a temperature gradient (either steady‐state or transient) while measuring the temperature response of the sample at one or more locations. The temperature data are combined with thermal models to extract the thermal conductivity using inverse parameter fitting methods.

Here, we introduce several commonly used methods for characterizing thermal conductivity, as summarized in Table 2. In the following sub‐sections, the methods are roughly organized by the time scale of analysis and the heat source type: steady state (Section 3.1: Reference Bar Method), transient with pulsed heat sources (Section 3.2: Time‐Domain Thermoreflectance; Section 3.3: Laser Flash Method; and Section 3.4: Transient Plane Source Method); and transient with periodic heat sources (Section 3.5: 1D Ångstrom Method; Section 3.6: 2D Laser Ångstrom Method; and Section 3.7: 3ω Method). Note that this selected set of techniques is non‐exhaustive, but encompasses a large fraction of the techniques used to analyze the materials of interest in this review.

**Table 2:** Summary of thermal conductivity measurement techniques for electrically insulating films and sheets.

Method	Measurement timescale	Typical form factor	Anisotropy resolution	Notes
**Miniaturized 1D reference bar** [86, 87] 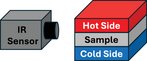	Steady‐state	Bulk solids (typically, micro‐ to milli‐scale)	Cut or fabricate samples with different orientations from a larger sample	Direct measurement of (through‐thickness) thermal conductivity. (Density and specific heat not required.) Requires a well‐characterized reference material of known thermal conductivity of similar magnitude to the sample. Minimum sample thickness limited by the diffraction limit of infrared temperature measurement (10's of microns).
**Thermoreflectance** 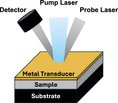	Transient (pulsed and/or periodic heat source)	Thin films (micro‐ to nano‐scale) on substrates and bulk materials coated with metallic transducer layer	Offset beam method for $k_{∥}$ anisotropy (e.g., [88]). Vary spot size or modulation frequency to separate $k_{∥}$ and $k_{⊥}$ (e.g., [89])	Commonly used for cross‐plane thermal conductivity and/or interfacial thermal resistance measurements. Typically requires knowledge of density, specific heat, and transducer properties to extract thermal conductivity. Modifications possible for in‐plane [90] and in‐plane vs. cross‐plane anisotropy [89]. Requires a optically smooth surface with a thin reflective coating on the sample.
**Laser flash** [91, 92] 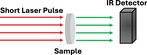	Transient (pulsed heat source)	Homogeneous solids (typically millimeter‐scale thickness)	Cut or fabricate samples with different orientations from larger sample or adapters available for in‐plane conductivity [93, 94, 95, 96]	Common commercially available tool for measuring cross‐plane thermal diffusivity. Typically requires knowledge of density and specific heat to extract thermal conductivity. Requires a thin absorptive and opaque coating on the sample if the material itself is not opaque and/or absorptive.
**Transient Plane Source** [92, 97] 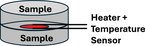	Transient (Pulsed heat source)	Bulk and smooth solids (typically, micro‐ to milli‐scale) with planar surfaces	Cut or fabricate samples with different orientations from larger sample	Commercially available tool for measuring $k_{⊥}$. Samples must be perfectly flat at the interface. Ideally, two ‘identical’ samples required for each test. Typically requires knowledge of density and specific heat to extract thermal conductivity.
**1D Ångstrom** 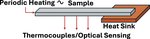	Transient (Periodic heat source)	Long, slender rods/strips (wires and fibers) or narrow films that can be suspended.	Cut or fabricate samples with different orientations from larger sample	Developed to evaluate in‐plane or axial thermal diffusivity of macroscale samples [98]. Now adapted to measure micro‐ to milli‐scale thin films and filaments leveraging infrared thermography, for instance [99]. Typically requires knowledge of density and specific heat to extract thermal conductivity. Sample must be IR opaque or coated with a high emissivity coating when leveraging infrared temperature sensing. May be sensitive to end effects, radiative losses, and deviations from 1D heat transfer.
**2D Laser Ångstrom** 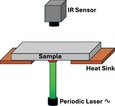	Transient (Periodic heat source)	Isotropic and anisotropic thin films and sheets that can be suspended over a gap.	Directly resolve in‐plane anisotropy with a single sample.	Developed to directly measure in‐plane thermal conductivity and thermal conductivity anisotropy (given knowledge of density and specific heat) [100]. Sample must be IR opaque or coated with a high emissivity coating when leveraging infrared temperature sensing. Sample must be “thermally thin” (generally, up to a few mm thick, depending on properties and heating frequency) [101].
**3** ω [102, 103, 104] 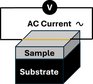	Transient (Periodic heat source)	Electrically insulating bulk materials and (suspended or supported) thin films with a patterned metal heater‐thermometer.	Modifications such as varying heater width on same sample to separate $k_{∥}$ and $k_{⊥}$ or suspending sample to promote in‐plane spreading.	Commonly used to measure $k_{⊥}$ [105], but can resolve $k_{∥}$ in certain configurations. Can also measure volumetric heat capacity by sweeping over appropriate frequency range. Generally requires planar samples with patterned heater line. Well suited to measuring supported and suspended films.

### 3.1 Reference Bar Method

To directly measure thermal conductivity, steady state techniques are generally required (as opposed to transient techniques, which are sensitive to the thermal diffusivity or thermal effusivity, requiring knowledge or measurement of the density and specific heat). The ASTM D5470 standard, often referred to as the conventional “reference bar” or “meter bar” apparatus [85], is a common steady‐state method to measure thermal conductivity. Briefly, the technique involves sandwiching the sample with unknown thermal conductivity between two materials of known thermal conductivity (a.k.a. the “reference bars”). Traditionally, point measurements of the temperature at several positions within the reference bar via thermocouples, for instance, enable quantification of the heat flux through the sample based on the linear temperature gradient and the known reference conductivity via Fourier's Law ($q^{\prime \prime }=-k_{ref}\;dT/dx)$. The measured temperature profiles in the reference bars are extrapolated to determine the temperature drop across the sample. The thermal conductivity of the sample is then calculated by using the heat flux (as measured from the reference region) and the temperature gradient across the sample, based on Fourier's law.

In the conventional method with thermocouples only instrumented within the reference bar, it is challenging to extract thermal conductivity independent of thermal interface resistances without testing multiple samples of different thickness. Thus, recent efforts have focused on integrating high‐resolution thermal imaging techniques with the reference bar approach to directly measure thermal conductivity and thermal interface resistance simultaneously [106, 107, 108].

The reference bar technique measures the thermal conductivity along the axis of the reference bar, which is typically the through‐thickness or cross‐plane thermal conductivity direction for thin films and sheets. Typical sample size requirements for this approach range from rectilinear samples with 1 cm × 1 cm cross‐section to 1 in diameter pucks with thicknesses from microns (thin films and sheets) to millimeters (bulk solids). Although the technique can be scaled to other form factors and dimensions, the thickness is often much smaller than the cross‐sectional dimensions in order to minimize convection losses. In order to measure “in‐plane” thermal conductivity of a sheet (or more generally, the 3 orthotropic components of thermal conductivity of a material), samples must be cut from a larger sample oriented such that the heat flux is directed parallel to the axis of interest. Thus, it is not suitable for measuring in‐plane thermal transport for very thin films.

### 3.2 Thermoreflectance

The thermoreflectance method is a non‐contact and non‐destructive optical technique used to measure the thermal properties of a material or a device based on the changes in the surface reflectance of the topmost layer due to changes in temperature. This versatile technique, originally developed for cross‐plane thermal conductivity measurements, has been adapted to measure thermal interface resistances and thermal conductivity anisotropy [90, 109, 110]. This method is typically used for solid and smooth materials with thicknesses spanning the range of microns to millimeters.

To briefly describe the method, generally, a short laser pulse (pulse duration from femtoseconds to nanoseconds) heats the surface of a sample, leading to a temperature rise and decay that can be measured using a (weaker) probe laser that monitors the change in reflectivity of the sample. Often, a transducer layer, consisting of a thin (∼50 nm) metal layer, is coated on the surface of a sample to confine heating to the surface and provide the desired optical reflection and absorption properties [111, 112]. Thermoreflectance measurements have recently been extended to the frequency‐domain where a continuous wave laser is modulated periodically and the transient temperature response is monitored [113, 114]. In transient thermoreflectance (TTR) and frequency‐domain thermoreflectance (FDTR), a continuous probe laser beam monitors the temperature response, while in time‐domain thermoreflectance (TDTR), both the pump and probe beams are pulsed and there is a controlled time delay between the pump and probe pulses to measure the time‐dependent thermal response. The reflected laser beam is typically measured by a photo‐diode sensor. In TDTR, a lock‐in amplifier measures the in phase and out of phase signal at the modulated frequency, while oscilloscopes or other data acquisition systems are typically used for TTR. The thermal response is then used to calculate the thermal properties of the material.

Most commonly, these techniques measure cross‐plane thermal conductivity and thermal boundary resistances of interfaces that are close to the exposed surface of the sample [115, 116]. But depending on the models used for data fitting and the sample configuration, thermoreflectance techniques can be used to extract numerous properties simultaneously including in‐plane and cross‐plane thermal conductivity, contact resistances (*R* 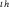) [116, 117], and/or volumetric heat capacity [118]. Care must be taken to design sample stacks for which the thermoreflectance system can resolve the properties of interest.

Typically, thermoreflectance experiments are carried out using a spot size larger than the thermal penetration depth of the sample, which causes the temperature gradient to be primarily in the cross‐plane direction. But it can be adapted to measure the in‐plane thermal conductivity of materials and thin films often by changing the relative diameters of the pump and probe beams or by scanning the probe beam laterally over the surface near pump beam [90]. For in‐plane measurements, the spot size of the heating beam is reduced such that the temperature gradient becomes three dimensional. Using two sets of measurements at the same modulation frequency, both the in‐plane and cross‐plane components of thermal conductivity can be measured [89]. While TDTR techniques are capable of measuring both the thermal conductivity as well as interfacial resistance, the physical experimental setup requirements including the laser systems and optics are complex.

### 3.3 Laser Flash Method

The laser flash technique employs non‐contact heating and sensing, and was introduced by Parker et al. in 1961 [119]. This method is typically used for homogenous solids (in the millimeter thickness range) and uses a pulsed optical heating source (such as a laser), in conjunction with an IR detector for fast and non‐invasive temperature sensing. Typically, a disk‐shaped sample is coated with a thin layer of highly emissive and absorptive graphite on both sides, which acts as a light absorber and an emitter for temperature sensing. The front side of the sample is uniformly heated with a pulsed light source, while the IR detector measures the temperature rise on the opposite side of the sample as a function of time. The transient temperature response is then used to fit the thermal diffusivity of the material. The faster the temperature rise on the rear side, the higher is the thermal diffusivity of the sample. In the simplest models to extract thermal properties, heat conduction is assumed to be one‐dimensional with no lateral heat loss, while more advanced data extraction techniques can include additional terms. Typically, the thermal diffusivity of the sample is directly proportional to the square of sample thickness and inversely proportional to the time required for the rear face to reach half of its maximum temperature rise. ASTM E1461 [92] specifies the requirements for the apparatus, test sample, and procedure for thermal diffusivity measurement using this technique. The density and specific heat of the materials must be measured separately to obtain the thermal conductivity from the measured data.

The laser flash method is capable of measuring thermal diffusivity over a wide temperature range, and it has several advantages over steady state methods including speed (usually only a few seconds for most solids) and the ability to use very small samples, typically 5‐12 mm in diameter [120, 121]. This method is accurate when the laser pulse is sufficiently short compared to the characteristic time of diffusion through the sample, but for very thin or high conductivity samples, the accuracy of the laser flash method is low [122, 123, 124].

This method is generally utilized to measure the cross‐plane thermal conductivity, but recent studies have also explored the extension of this technique for measuring in‐plane thermal diffusivity of films [93, 94, 95, 96]. In this modified technique, instead of uniformly heating one of the surfaces, a ring shaped heating profile is applied to one side of the sample, and the transient temperature rise of center of the opposite side of the sample is measured to fit for the in‐plane thermal diffusivity. This analysis assumes isotropic in‐plane thermal conduction.

### 3.4 Transient Plane Source Method

The transient plane source (TPS) method, also called the hot disk method, uses a thin metal strip or disk embedded in an electrically insulating film as a heat source and temperature sensor. The heat source is sandwiched between two samples of the material under test [97]. The samples are insulated on all other sides. After applying current to the heater, the temperature rises, and is monitored to determine the thermal properties of the samples. The heating is limited to a short period of time (typically a few seconds) to ensure that the samples can be approximated as semi‐infinite throughout the data acquisition process. The thermal conductivity of the material is obtained by linearly fitting the experimental temperature rise (based on the electrical resistance of the sensor) against a dimensionless, time‐dependent geometric function, D(τ), which accounts for the spatial integration of the heat source. The thermal conductivity is inversely proportional to the slope of this linear regression. This method is typically used for measuring the cross‐plane thermal conductivity of bulk solids with thicknesses spanning the microns‐to‐millimeter range. One limitation of the TPS method is that both samples must have a perfectly planar surface to maintain good thermal contact with the heater‐sensor. Generally, measurement errors in TPS arise from the non‐uniform thermal contact resistance between the sensor and the samples, the thermal inertia of the sensor, inaccuracies in the measurement of the power input, and the electrical resistance change of the heater‐sensor disks [122, 125].

### 3.5 1D Ångstrom Method

The traditional Ångstrom method has been used widely for over 150 years for the characterization of thermal diffusivity of films and long and slender 1‐D samples [98]. The measurement principle of this technique is to periodically heat one end of the sample and to measure the time varying temperature response of the sample at two or more locations away from the heat source. At the macroscale, this method is largely unaffected by convection and radiation losses, although microscale versions often require vacuum conditions [99, 126, 127]. The analysis involves fitting of the experimental temperature measurements to a one‐dimensional heat transfer model to calculate thermal diffusivity. A few recent studies have focused on making this technique more versatile, accurate, and scalable to smaller sample dimensions with non‐contact infrared microscopy‐based measurement of temperatures [99, 128, 129]. A tuning parameter in this technique is the frequency of periodic heating: adjusting the heating frequency can result in precise measurements of materials with widely varying thermal conductivities (spanning several orders of magnitude) [98, 130]. The conventional Ångstrom method utilized physical attachment of thermocouples for local temperature measurement. But for thin samples, this can cause significant heat losses, leading to large measurement uncertainty. Recently, Hahn et al. demonstrated an IR‐enhanced version of this measurement technique that enabled measurement of micro‐scale as well as semi‐transparent polymeric materials [99]. For a pair of discrete spatial locations along the propagation path, the thermal diffusivity (α) scales linearly with the thermal modulation frequency (ω) and the square of the difference between the two points $\left[{\left(x_{2}-x_{1}\right)}^{2}\right]$, and scales inversely with both the spatial phase lag ($\phi_{2}-\phi_{1}$) and the logarithmic amplitude decay $\left[\ln(A_{1}/A_{2})\right]$ evaluated between those two points. In the context of adapting this technique with high‐resolution infrared (IR) thermography, where continuous temperatures can be acquired at multiple spatial locations, the thermal diffusivity is robustly extracted via linear regression by mapping the relationship between the terms containing the phase shift and the logarithmic amplitude attenuation as a strictly linear function of spatial position $\left[{\left(x-x_{1}\right)}^{2}\right]$. Typically, the Ångstrom‐based methods are limited to measurements along a single direction and require multiple strips to determine the anisotropy of a material along different directions.

### 3.6 2D Laser Ångstrom Method

The two‐dimensional (2D) Laser Ångström method is a recent technique developed to measure the orthotropic in‐plane thermal conductivity of thin films and sheets exhibiting in‐plane anisotropy [100, 101, 131]. It extends the traditional Ångstrom method, originally designed for one‐dimensional thermal conduction in rod‐like samples, to two‐dimensional conduction in 2D films and sheets. This non‐contact, non‐invasive technique employs periodic laser heating at the center of the sample's back surface, which is suspended over a metallic heat sink with a circular opening. Transparent samples may require a thin, thermally black metallic disk on the underside to act as an absorber. The periodic heating induces spatio‐temporal temperature oscillations, which are recorded on the top surface using infrared (IR) imaging once the specimen reaches a steady‐periodic thermal state. These temperature oscillations should fundamentally satisfy the two‐dimensional heat diffusion equation (considering convective losses), which are then Fourier‐transformed into the frequency domain. Post‐processing of the amplitude and phase lag of the temperature response at different frequencies allows simultaneous extraction of orthotropic in‐plane thermal conductivities using least squares fitting algorithms, independent of heat input or peripheral boundary conditions. Recent work has further demonstrated that machine learning algorithms can be employed to accurately extract in‐plane thermal conductivities even from noisy experimental data [132].

This method relies on the assumption that temperature gradients across the specimen thickness are negligible compared to in‐plane gradients, which can be achieved by choosing a heating frequency such that the thermal penetration depth exceeds the sample thickness. Its ability to simultaneously measure both principal in‐plane thermal conductivities with minimal sample perturbation makes it especially suitable for thin films and in‐plane anisotropic materials.

### 3.7 3ω Method

The 3ω method is one of the most widely employed electro‐thermal techniques for measuring the thermal conductivity of both bulk materials and thin films [105, 133, 134]. The method typically involves depositing a thin metal line on the sample surface to serve as both heater and temperature sensor. An alternating current (AC) passed through this heater at a frequency ω generates Joule heating (∝ square of current), producing temperature oscillations at twice the frequency of the applied current (2ω). These temperature oscillations result in variation of the electrical resistance of the line heater at the same frequency (2ω). Consequently, the voltage signal (product of current and resistance) contains frequency components at ω and 3ω, with the latter being extracted using a lock‐in amplifier. Analysis of the 3ω voltage component allows extraction of the sample's thermal conductivity through well‐established models. In the simplest case for anisotropic solids, the geometric mean of the in‐plane and cross‐plane thermal conductivity scales directly with the cube of the excitation current ($I^{3}$), the ambient‐temperature electrical resistance of the heater ($R_{0}$), and the temperature coefficient of resistance (α), while exhibiting an inverse proportionality to the physical length of the line heater (L) [134]. At a fixed modulation frequency, the thermal conductivity is inversely proportional to the absolute out‐of‐phase voltage component (Y or $V_{3\omega ,\text{out-of-phase}}$). When considering multiple frequencies, the thermal conductivity is inversely proportional to the derivative of the in‐phase (X or $V_{3\omega ,\text{in-phase}}$) voltage component with respect to the natural logarithm of the modulation frequency ($\partial V_{3\omega }/\partial \ln\omega$).

The 3ω method has been adapted to measure the thermal conductivity of anisotropic bulk samples and thin films [102, 103, 104]. Despite its versatility, the 3ω method has several limitations. Accurate measurements require high‐quality deposition of the metal heater with well‐controlled dimensions and thermal contact. For thin films, the method assumes negligible heat loss to the surrounding environment, which can be difficult to achieve in practice. Furthermore, the technique is most sensitive to in‐plane thermal conductivity when the heater width is comparable to the film thickness, limiting its applicability to very thin or highly anisotropic films. At very low or high thermal conductivities, signal‐to‐noise ratio and thermal penetration depth constraints may introduce additional uncertainties [135]. Finally, interpretation of the 3ω signal often relies on simplified analytical models that may not fully capture complex geometries or multilayer structures.

## 4 Thermal Conductivity of Dielectric Materials: Trends and Discussion

This section examines trends in the anisotropic thermal conductivity of different classes of electrically‐insulating materials, including those with intrinsic and engineered anisotropy. When discussing anisotropy, we focus on the in‐plane versus cross‐plane conductivity variations as most of the relevant materials are relatively isotropic within the various in‐plane directions. For thin sheets and films, the cross‐plane direction corresponds to transport through the thickness (typically the smallest dimension) of the sample, while the in‐plane direction refers to heat flow along the plane of the material. For intrinsically anisotropic crystals such as Boron Nitride (BN), Aluminum Nitride (AlN), Silicon Nitride (Si_3_N_4_), and sapphire (Al_2_O_3_), these terms describe conduction parallel and perpendicular to specific crystallographic axes. For instance, for BN, in‐plane corresponds to the basal plane, whereas cross‐plane refers to the direction perpendicular to the basal plane. In other intrinsic materials, the in‐plane and cross‐plane directions similarly correspond to transport parallel and perpendicular to the *c*‐axis. The anisotropy ratio (AR) is defined here as the ratio of in‐plane to cross‐plane thermal conductivity. All data used in the following analyses is tabulated in Supporting Information S1. A persistent challenge in the field is the incomplete reporting of both in‐plane and cross‐plane conductivities, as well as film thicknesses. This lack of comprehensive data complicates systematic assessment of anisotropy and highlights the need for more complete characterization in future studies.

Figure 2 presents cross‐plane versus in‐plane thermal conductivity for electrically insulating materials. For reference, the thermal conductivities of benchmark materials are also included although not all of these reference materials satisfy the electrically insulating requirements of this review. For low‐conductivity, isotropic materials (i.e., AR = 1), Teflon (T) serves as a baseline with a thermal conductivity of ∼0.3 W $m^{-1}$ $K^{-1}$ [100]. At the other extreme, copper (C) and single‐crystal diamond (D) define the high‐conductivity regime, with values of ∼400 W $m^{-1}$ $K^{-1}$ [150] and ∼2000 W $m^{-1}$ $K^{-1}$ [151], respectively. As a reference anisotropic material, graphite (G) occupies the high‐conductivity AR regime with in‐plane conductivity of ∼2000 W $m^{-1}$ $K^{-1}$ and cross‐plane conductivity of ∼10 W $m^{-1}$ $K^{-1}$, resulting in an AR of ∼200 [152].

**Figure 2:**
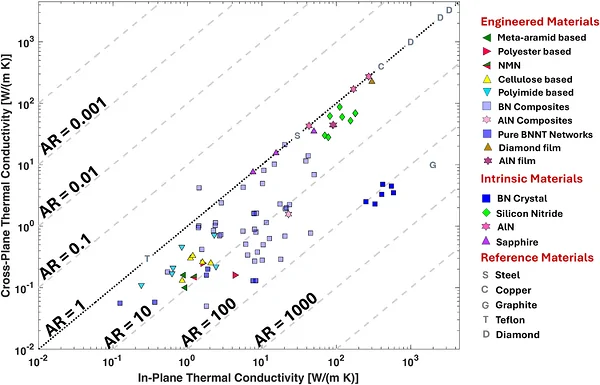
Cross‐plane versus in‐plane thermal conductivity for engineered materials, intrinsic materials, and reference materials. Most materials exhibit anisotropy ratios between 1 and 10, with both in‐plane and cross‐plane thermal conductivities typically below 100 W $m^{-1}$ $K^{-1}$. Exceptions include boron nitride crystals and graphite, which show anisotropy ratios exceeding 100, and materials such as humidity‐resistant sheets (polyester‐based), aluminum nitride, certain BN composites, and pure BNNT networks, which display anisotropy ratios in the range of 10–100. In general, the in‐plane thermal conductivity of the engineered materials is higher than cross‐plane thermal conductivity due to fiber orientation effects introduced during fabrication, which align thermally conductive fillers along the in‐plane direction. Data from references [11, 18, 19, 21, 29, 30, 31, 32, 33, 34, 35, 44, 46, 49, 55, 56, 57, 58, 59, 60, 61, 62, 64, 68, 70, 72, 73, 74, 75, 76, 78, 79, 80, 81, 82, 83, 84, 88, 100, 131, 136, 137, 138, 139, 140, 141, 142, 143, 144, 145, 146, 147, 148, 149, 150, 151, 152, 153, 154, 155, 156].

In general, most materials exhibit higher in‐plane thermal conductivity compared to cross‐plane directions, resulting in anisotropy ratios greater than 1. This effect is particularly pronounced in engineered materials, including polymers, polymer‐matrix composites (PMCs), and ceramic‐matrix composites (CMCs), where processing methods intentionally align thermally conductive fillers or fibers along the in‐plane direction (see Section 2.2). For example, most engineered materials lie near an AR of 10, whereas intrinsic materials have an AR generally much less than 10, with the exception of *h*‐BN which has an AR of ∼100.

In terms of thermal conductivities, a clear hierarchy is observed. Pure polymers consistently occupy the low‐conductivity regime, intrinsic crystalline solids define the high end, and composites typically span the intermediate range depending on filler type, concentration, and orientation. Notably, pure BNNT networks possess lower thermal conductivity especially in the through‐plane direction and is likely due to their porous microstructures [46]. In contrast, highly engineered composites approach high in‐plane thermal conductivity through careful microstructural design. Such improved thermal conductivities are usually achieved with the help of adopting functionalization and alignment strategies that help in reducing the interfacial resistance between the fillers and the matrix and aligning the thermally conductive fillers. For instance, Sun et al. [61] achieved exceptionally high in‐plane thermal conductivity (45.15 W $m^{-1}$ $K^{-1}$ with only 37.5 wt.%) in BNNS (functionalized by PDA)/PBO nanofiber films by inducing a nacre‐like lamellar structure and strong interfacial interactions through deprotonation and hot‐pressing. Yu et al. [60] showed that uniform dispersion of *h*‐BN into a polymer matrix via ball milling, followed by coagulation in ethanol, vacuum drying, and hot pressing, can produce flexible composite films with well‐distributed fillers and an enhanced thermal conductivity of 50.3 W $m^{-1}$ $K^{-1}$, although at a much higher loading of 95 wt.%. However, it is important to note that the best‐performing thermal management material does not necessarily possess the largest thermal conductivity, but rather the anisotropy ratio is a critical factor when precise control of thermal transport is required. The target anisotropy ratio is governed by the specific heat routing requirements of the system. For applications requiring lateral hotspot spreading and simultaneous vertical thermal shielding, a high anisotropy ratio (characterized by higher in‐plane thermal conductivity and lower cross‐plane conductivity) is optimal. In contrast, when the objective is direct heat sinking, a lower anisotropy ratio with maximized cross‐plane thermal conductivity is required to minimize the vertical thermal resistance.

The distribution of anisotropy ratios underscores the striking differences among material classes. Bulk *h*‐BN crystals exhibit the highest ratio in the dataset, with an average AR of approximately 124 ± 34 [21], comparable to graphite (∼200 [152]), due to the similarities in their bonding structures. In contrast, other intrinsic materials such as AlN, Si_3_N_4_, and sapphire show relatively weak anisotropy, with ratios below 3. Most engineered materials fall within the range of 1–10, although select BN and AlN‐based composites can reach ratios of 10–100 depending on their processing methods. Among engineered materials, the maximum observed anisotropy ratio achieved thus far are 61.72 for an electrospun BNNS‐FPU composite [68] and 62 for a porous BNNT fabric [46].

Interestingly, a small number of engineered materials exhibit anisotropy ratios close to unity. These systems are deliberately engineered to enhance cross‐plane transport rather than in‐plane performance, often through vertical alignment strategies. For example, Xiao et al. [75] employed a specialized vacuum filtration method to vertically align SiC nanowire/functionalized BNNS frameworks, which were subsequently impregnated with epoxy resin, resulting in an anisotropy ratio of 0.34 [75]. Other methods include ice templating (or freeze casting), in which directional freezing of a solvent induces vertical alignment of filler particles. Huang et al. [77] used this to fabricate *h*‐BN‐PVA composites with an anisotropy ratio of 0.95.

The vast majority of materials reviewed here display anisotropy only between in‐plane and cross‐plane directions, with little or no in‐plane anisotropy. However, there are a few materials like Temprion, which show strong in‐plane anisotropy, with thermal conductivity of ∼40 W $m^{-1}$ $K^{-1}$ along the fiber axis and <1 W $m^{-1}$ $K^{-1}$ in the transverse direction [100]. Similarly, the Fluorinated Ethylene Propylene (FEP) layer in the Kapton FN laminate also shows in‐plane anisotropy of around 2.2 between the machine direction (0.24 W $m^{-1}$ $K^{-1}$) and the transverse direction (0.11 W $m^{-1}$ $K^{-1}$) [157]. In addition, there is growing interest in the development of polymer fibers and composites that exhibit in‐plane thermal anisotropy, particularly for dielectric, flexible, and wearable heat‐spreading applications [158]. Such materials inherently possess directional thermal properties arising from chain alignment and orientation. For instance, highly oriented polyethylene films [159] and fabrics constructed from ultra‐high molecular weight polyethylene fibers [160] have demonstrated significantly enhanced in‐plane thermal conductivity along the fiber direction, highlighting the potential for tunable anisotropic heat transport in polymer‐based systems. A common strategy to induce high directional thermal transport involves the extrusion or spinning of polymer/BN filler mixtures followed by post‐processing treatments like hot pressing, which preferentially align the BNNTs along the processing direction. For instance, this approach has been successfully implemented by Park et al. [161] using an epoxy matrix via micro‐nozzle extrusion and by Terao et al. [70] using electrospun polyvinyl alcohol (PVA), achieving distinct in‐plane thermal anisotropy ratios of 2.53 and 2, respectively. Similarly, Ohgoshi et al. [162] demonstrated that aligning magnesia (MgO) nanofibers (which are intrinsically isotropic) yields multidimensional anisotropy, establishing distinct variations both within the in‐plane profile (12.7 W $m^{-1}$ $K^{-1}$ along the fiber direction versus 7 W $m^{-1}$ $K^{-1}$ perpendicular to it) and between the in‐plane and cross‐plane axes (12.7 and 7 vs. 1 W $m^{-1}$ $K^{-1}$). These cases represent the configuration illustrated in Figure 1(e). Niu et al. [163] also demonstrated the anisotropy by modifying the filler network architecture and fabricating a vertically aligned boron nitride film/silicone rubber (V–BNF/SR) composite via a precise stacking‐cutting method. This structural arrangement yielded a clear in‐plane biaxial anisotropy ratio of 1.94 ($k_{x}=0.867$ vs. $k_{y}=0.447$ W $m^{-1}$ $K^{-1}$), alongside an extremely low AR of of <0.05 driven by a remarkably high through‐plane thermal conductivity of ∼19 W $m^{-1}$ $K^{-1}$.

From an application perspective, the desirable anisotropy ratio depends strongly on context: high in‐plane conduction is advantageous for lateral heat spreading in electronic substrates, while enhanced through‐plane transport is critical for vertical heat dissipation between materials or components. Higher anisotropy ratios give more control over the direction of thermal transport by acting as a thermal insulator in one direction and a thermal conductor in the orthogonal direction. Adding further control are the few materials that exhibit in‐plane anisotropy and another dimension of thermal management. This wide selection of thermal transport properties presents the diversity of design strategies observed in thermal management composites. As Figure 2 suggests, however, the ultimate benchmark for the highest thermal conductivity of electrically insulating materials remains intrinsic crystalline bulk solids.

### 4.1 Impact of Thickness

Figure 3 shows the in‐plane and cross‐plane thermal conductivity as a function of material thickness and Figure 4 shows the anisotropy ratio as a function of thickness for these materials. In general, there are no clear trends of the thermal conductivity and anisotropy ratio with material thickness that span across different types of materials. Most reported engineered materials fall within the practically relevant thickness range of 10–1000 µm. This range of thicknesses is particularly relevant to thermal management layers in device architectures for modern electronic devices.

**Figure 3:**
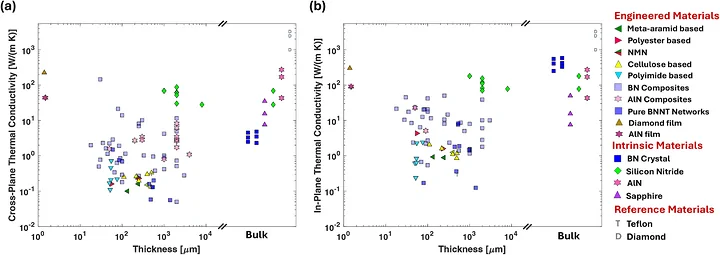
(a) Cross‐plane thermal conductivity versus thickness and (b) in‐plane thermal conductivity versus thickness for electrically insulating materials, compared with bulk values of the corresponding pure crystals. For BN‐based composites, the cross‐plane thermal conductivity often exceeds that of bulk BN crystal due to alignment of fillers and improved interfacial coupling, whereas the in‐plane thermal conductivity cannot surpass the pure crystalline BN. This anisotropy enhancement is not observed in AlN composites, where the intrinsic conductivity is already high and isotropic; polymer addition reduces conductivity in both directions. Silicon nitride films typically follow bulk values. Extremely thin AlN and diamond films show reduced conductivity relative to bulk due to phonon scattering. Data from references [11, 18, 19, 21, 29, 30, 31, 32, 33, 34, 35, 44, 46, 47, 48, 49, 55, 56, 57, 58, 59, 60, 61, 62, 68, 70, 72, 73, 75, 76, 78, 79, 80, 81, 83, 84, 88, 100, 136, 137, 139, 140, 141, 143, 144, 145, 147, 148, 149, 150, 151, 152, 153, 154, 155, 156, 157, 164, 165, 166, 167, 168, 169, 170, 171, 172, 173, 174, 175, 176, 177].

**Figure 4:**
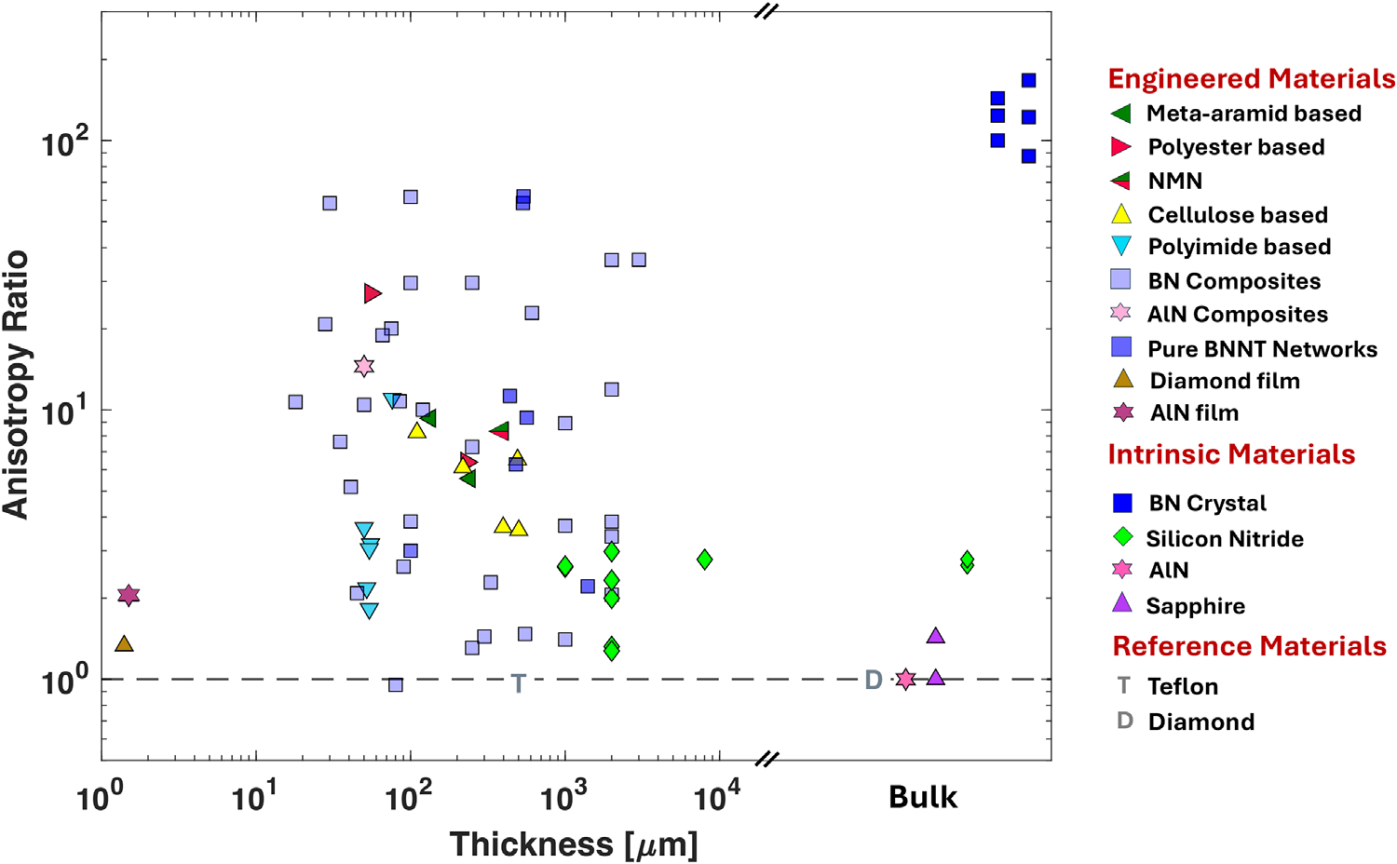
Anisotropy ratio (in‐plane/cross‐plane thermal conductivity) as a function of thickness for various electrically insulating materials, benchmarked against bulk crystalline values. With increasing thickness, most materials exhibit anisotropy ratios approaching unity due to phonon scattering and boundary effects. Bulk Boron Nitride and Silicon Nitride crystals maintain anisotropy ratios >1, while polymeric composites span a broad range (1–100). A pure BNNT network demonstrates a high anisotropy ratio (63.5), closely approaching that of bulk BN crystal (87.5). Data from references [11, 18, 19, 21, 29, 30, 31, 32, 33, 34, 35, 44, 46, 47, 48, 49, 55, 56, 57, 58, 59, 60, 61, 62, 68, 70, 72, 73, 75, 76, 78, 79, 80, 81, 83, 84, 88, 100, 136, 137, 139, 140, 141, 143, 144, 145, 147, 148, 149, 150, 151, 152, 153, 154, 155, 156, 157, 164, 165, 166, 167, 168, 169, 170, 171, 172, 173, 174, 175, 176, 177].

Note that for crystalline materials, the thickness introduces another dimension to this comparison. In the sub‐micron and thin‐film regime (typically less than 5 µm), the size effects and phonon boundary scattering dominate crystalline materials. For instance, thin diamond films (1.4 µm thick), while still highly conductive, exhibit substantial reductions in thermal conductivity relative to bulk values due to size effects and boundary scattering [149]. In contrast, AlN thin films show a less pronounced conductivity degradation with decreasing thickness [148]. This resilience suggests that AlN preserves more of its bulk‐like transport characteristics at reduced dimensions, making it especially attractive for microelectronic applications. Some reports quantify thermal transport in thin AlN films with values comparable to the higher end of bulk AlN [178, 179], but only one component of the thermal conductivity tensor is measured in these studies.

As thickness increases into the microscale regime, these intrinsic phonon size effects gradually become negligible. Instead, transport transitions to being dominated by composite microstructural features, such as filler dispersion, local orientation, and interfacial boundaries, rather than purely geometric restrictions on the phonon mean free path. The exact thickness at which the size effects stop influencing the thermal properties (due to phonon‐boundary scattering) depends on the type of material and temperature.

### 4.2 Comparison of Engineered and Bulk Materials

While the exact thickness does not specifically impact thermal conductivity and anisotropy ratio, a clear trend emerges when comparing engineered composites with their bulk counterparts.

For instance, Boron nitride (BN) composites typically have lower in‐plane thermal conductivity than bulk‐crystalline *h‐*BN, even at filler loadings exceeding 80 vol.% [60], as is expected of typical polymer composites with the matrix and filler exhibiting widely different properties. In particular, this reduction stems from severe in‐plane phonon scattering at recurring polymer‐filler interfacial bottlenecks that interrupt the highly conductive basal plane. Interestingly, several BN‐based composites exhibit comparatively higher cross‐plane conductivity than the cross‐plane direction for bulk BN (perpendicular to the basal plane of the crystal). This enhancement occurs because functionalized polymer interfaces and percolated nanofiller networks provide lower thermal resistance than the weak van der Waals interactions between the layers of bulk crystalline BN. This mechanism has been demonstrated across various studies [77, 143, 145], where surface functionalization of the BN nanofillers improves their dispersion within the polymer matrix and strengthens interfacial interactions via hydrogen bonding or polarization, ultimately enabling superior phonon vibration matching, and better thermal transport. This suggests that interfacial architecture and filler alignment may preferentially enhance cross‐plane transport or simply that the matrix material increases thermal conductivity orthogonal to the filler alignment direction. This underscores the subtle interplay between microstructural design and phonon scattering pathways. Given the large number of reported materials with BN fillers, Section 4.3 discusses these composites in more detail.

Another example is the case of aluminum nitride (AlN), which differs in the trends observed for BN. Both in‐plane and cross‐plane conductivities of AlN composites remain well below those of bulk crystalline AlN, likely due to intrinsic bonding characteristics of AlN. Its strong, but weakly dispersive, phonon spectrum makes it particularly vulnerable to defect and interface scattering, preventing composite systems from approaching crystalline performance.

As noted previously, the anisotropy ratio is also important for strategic thermal management, rather than solely the magnitude of thermal conductivity. Bulk crystalline materials, such as BN and Si_3_N_4_, retain anisotropy ratios substantially greater than one, highlighting the persistence of their intrinsically anisotropic bonding and phonon dispersions. In contrast, polymer‐based composites span a broad range of anisotropy values (≃1–100), underscoring the variability introduced by filler morphology, dispersion, and interfacial thermal resistance. A particularly striking case is that of a pure BNNT network, which demonstrates an anisotropy ratio of approximately 62 [46], closely approaching that of bulk BN crystal (124 [18]). This emphasizes the strong intrinsic anisotropy of BN‐based nanostructures and the potential to harness this for directional heat transport applications through proper engineering and material design. This also demonstrates the importance of considering the anisotropy ratio and not solely relying on the magnitude in thermal conductivity to evaluate a material's performance for thermal management applications. Among BN composites, higher anisotropy ratios at larger thicknesses can be attributed to enhanced filler alignment and reduced boundary limitations, which together amplify in‐plane conduction relative to cross‐plane pathways. This behavior illustrates how microstructural optimization at mesoscopic length scales can preserve or even enhance anisotropic transport, in contrast to the homogenization effects often observed in thinner films or less ordered composites.

### 4.3 Thermal Conductivity of BN Composites: Impact of Filler Loading Fraction

Among the thermally anisotropic, electrically insulating materials, BN‐based composites are the most widely researched class of materials for thermal management. BN‐based composites attract particular attention because they combine the intrinsically high anisotropy and electrically insulative properties of BN (listed in Table 1) with the tunability of composite architectures, making them central to many thermal management strategies. Moreover, the extensive literature available on BN fillers provides a rich dataset for systematic comparison across different processing methods and loading levels. As a result, the following section will discuss the trends and properties of BN‐based composite materials.

Figure 5 presents (a) cross‐plane thermal conductivities, (b) in‐plane thermal conductivities, and (c) anisotropy ratios of BN composites as a function of loading fraction. The loading fraction is most appropriately expressed as a volume fraction, defined as the ratio of filler volume to the total composite volume. However, many reports instead present filler content in terms of weight fraction. To enable consistent comparison, reported weight fractions were converted to volume fractions using the known densities of *h‐*BN (2.1 g ${\mathrm{cm}}^{-3}$) and the corresponding matrix materials, providing a consistent representation of filler concentration effects. The conversion from weight percentage (wt.%) to volume percentage (vol.%) follows:

(1) $$vol.\%=\frac{\frac{wt.\%}{\rho_{BN}}}{\frac{wt.\%}{\rho_{BN}}+\frac{\left(100-wt.\%\right)}{\rho_{mat}}},$$

where $\rho_{BN}$ and $\rho_{mat}$ are the densities of the *h‐*BN filler and the matrix, respectively. For studies in which thermal conductivity data were only provided in graphical form, individual points at specific loading fractions were extracted using WebPlotDigitizer [180], thereby enabling direct comparison across different reports.

**Figure 5:**
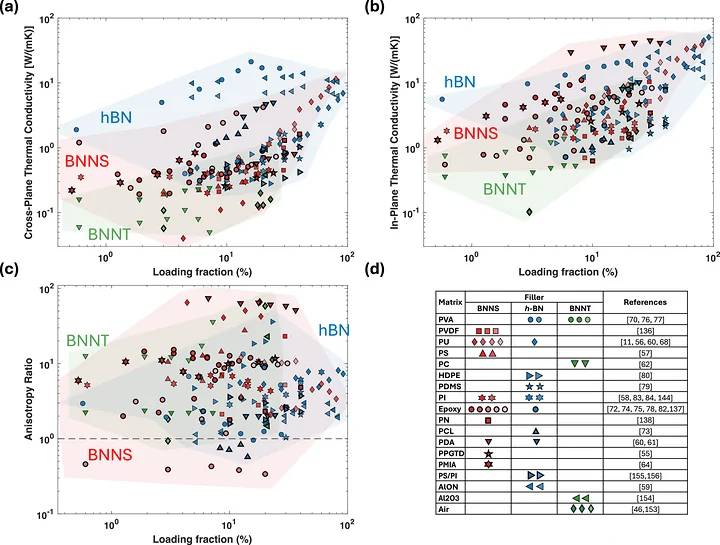
(a) Cross‐plane thermal conductivities, (b) in‐plane thermal conductivities, and (c) anisotropy ratio of BN composites as a function of loading fraction (or solid volume fraction) for the material combinations documented in panel (d). Ceramic‐based composites typically exhibit higher thermal conductivities and porous BNNT networks (no solid matrix) occupy the lower end. Polymer‐based composites occupy a broad range of conductivities. Thermal conductivity typically increases with loading fraction with deviations arising from agglomeration, voids, anisotropic alignment, and interfacial resistance. BNNT‐based composites show lower conductivities, while *h‐*BN and BNNS‐based systems achieve higher values. No clear trend is observed for anisotropy ratios: for most materials, it varies irregularly, while in some cases it increases with loading and, in a few cases, it first increases then decreases. Variations reflect the influence of filler alignment, network formation, and matrix interactions on directional thermal transport [11, 46, 55, 56, 57, 58, 59, 60, 61, 62, 64, 68, 70, 72, 73, 74, 75, 76, 77, 78, 79, 80, 81, 82, 83, 84, 136, 137, 138, 144, 153, 154, 155, 156].

There are three types of BN fillers highlighted in Figure 5: *h*‐BN particles (blue), BNNSs (red), and BNNTs (green). Each filler type has been highlighted with a different color to create a shaded region and visualize the material's impact on thermal conductivity. BNNT‐based composites consistently occupy the lower end of both in‐plane and cross‐plane thermal conductivities at a particular loading fraction. However, there are fewer studies aimed at improving the thermal conductivity of BNNT‐based composites. This is largely due to challenges in processing and integrating BNNTs into composite materials: their high aspect ratio and entangled morphology make uniform dispersion difficult, and it is particularly challenging to incorporate them at high loading fractions without forming agglomerates or voids [181]. Depending on the application and desired thermal management strategy, the low cross‐plane thermal conductivity of BNNT composites may offer advantages like improved anisotropy ratios which will be discussed later. In contrast, *h‐*BN and BNNS‐based composites tend to occupy the higher end of the thermal conductivity. This reflects their easier processability, resulting in higher loadings, more continuous thermal networks, and in some cases better alignment. Composites made with *h‐*BN particle fillers on average, have larger cross‐plane thermal conductivities than BNNS and BNNT fillers. This is likely due to the lower aspect ratio of *h‐*BN particles that reduces alignment compared to BNNS and BNNT fillers. As a whole, BN‐based composites display a mixed spectrum of tunable conductivities, with filler type and loading playing a dominant role.

Another important aspect to consider is the matrix composition. The majority of reported BN‐based composites employ polymer matrices, reflecting their ease of processing, low cost, and widespread use in electronic packaging. A wide variety of polymer matrix chemistries have been utilized and differences in polymer chemistry influence the filler–matrix interactions that can strongly influence thermal transport. This variation in polymer matrix chemistry may also be contributing to the broad spread of thermal conductivities observed. In contrast, only a limited number of ceramic matrix composites have been reported, such as systems incorporating aluminum oxynitrile (AlON) [59] and Al_2_O_3_ [154] as the matrix, which offer improved thermal and mechanical stability over polymer matrices but are more challenging to process. A third category is represented by air‐based systems, exemplified by pure BNNT networks [46], where the absence of a dense matrix allows the intrinsic anisotropy of the BN nanostructures to dominate the transport behavior. Together, these three classes of composite matrix materials highlight the diverse strategies pursued to leverage anisotropy properties of BN within composite architectures.

In most BN‐based composites, the thermal conductivity increases with filler loading due to the formation of more continuous heat‐conducting networks. For polymer matrices, the largest enhancement for the in‐plane thermal conductivity was reported to be approximately 8900% (from 0.23 to 20.47 W $m^{-1}$ $K^{-1}$) in PU/BNNS composites [56] at a loading fraction of 36.4 vol.%. In contrast, the maximum improvement in the cross‐plane thermal conductivity, approximately 1500% (from 0.15 to 2.42 W $m^{-1}$ $K^{-1}$), was achieved in epoxy–BNNS composites fabricated via ice templating and infiltration at a loading fraction of 9.3 vol.% [74]. Although there is a general trend of increased thermal conductivity with loading fraction, several factors must be considered to avoid deviations from this trend:

- **Filler agglomeration and void formation**: At high loadings, excessive BN fillers can be difficult to disperse in a solvent or matrix resulting in agglomeration. The formation of aggregates prevents a homogeneous distribution within the matrix leading to internal voids and reduced alignment which disrupts phonon transport and reduces thermal conductivity. This in particular influences the in‐plane thermal conductivity more than the cross‐plane thermal conductivity. The agglomeration effects and the resulting decrease in thermal conductivity were observed in [61, 68, 73, 76].
- **Matrix–filler interactions**: Poor wettability or weak interfacial bonding between filler and matrix can exacerbate void formation and improper mixing, leading to localized insulation by air pockets and a decrease in thermal transport. This was observed by Zhang et al. [76]. Such weak coupling also introduces significant interfacial thermal resistance (Kapitza resistance) due to phonon mismatch at the interface. Consequently, phonon scattering at discontinuous filler–matrix boundaries becomes more pronounced, further limiting phonon‐mediated thermal transport.
- **Percolation and optimal loading points**: Some systems exhibit a peak in conductivity at intermediate loadings (e.g., 25 wt.%) corresponding to percolation thresholds, after which further increases in filler content can slightly reduce conductivity due to diminished alignment or network disruptions. This was observed by Wilk et al. [59] and Huang et al. [77].

Collectively, these observations highlight that achieving high thermal conductivity in BN composites is not solely a matter of increasing filler content but requires careful control of filler dispersion, alignment, and matrix–filler interactions. Furthermore, these principles are broadly applicable to non‐BN fillers as well. Achieving superior thermal transport ultimately relies on selecting optimal filler–matrix pairings, implementing strategies to enhance dispersion, engineering robust matrix–filler bonding, and aligning them appropriately to enhance the thermal transport in a specific direction.

Figure 5(c) presents the anisotropy ratio of BN‐based composites as a function of filler loading fraction. Unlike thermal conductivity, no consistent trends are observed: for most materials, the anisotropy ratio varies irregularly, sometimes increasing, decreasing, or remaining nearly constant with increasing loading. In certain data sets, the anisotropy ratio shows a monotonic increase with filler fraction, while in other cases it first increases and then decreases. This rise‐and‐fall can be explained by the agglomeration effects and percolation thresholds. At low‐to‐moderate volume fractions, processing‐induced shear aligns high‐aspect‐ratio BN fillers in the plane, enhancing in‐plane phonon percolation and steadily increasing the AR. However, surpassing an optimal loading threshold triggers severe spatial crowding, which induces filler agglomeration and destroys this directional alignment. Concurrently, poor matrix‐filler wettability may promote void formation, introducing isotropic air pockets that heighten the interfacial thermal resistance. Because this defect‐induced phonon scattering disrupts long‐range ballistic transport along the highly optimized in‐plane pathways far more severely than the already restricted cross‐plane paths, the AR undergoes a distinct decline at maximum loadings, thus completing the characteristic “rise‐then‐fall” trajectory.

The anisotropy ratio can vary from nearly 100 to less than 1 highlighting the tunability of BN‐based composites. This scattered behavior reflects the complex interplay between filler alignment, network connectivity, and matrix interactions, highlighting that anisotropy is highly sensitive to microstructural details rather than filler content alone. This is an important consideration when designing composite materials for thermal management; simply maximizing thermal conductivity may not result in the best thermal management material, but rather the optimization of thermal conductivity in each direction should be used to tailor the anisotropy ratio desired for the application. For example, in Figure 5(a) and 5(b), BNNT composites tend to have some of the lowest values for cross‐plane and in‐plane thermal conductivity. However, when considering the anisotropy ratio in Figure 5(c), the BNNT composites have some of the highest thermal anisotropy and depending on the application, may be desirable. This behavior is driven by the unique morphology of BNNTs. These 1D nanotubes are highly prone to nanotube entanglement, creating disordered localized meshes that restrict long‐range axial phonon propagation and reduce the thermal conductivity. Concurrently, this entangled network introduces substantial internal porosity, acting as an acoustic mismatch boundary that severely insulates the cross‐plane direction. By driving cross‐plane thermal conductivity to very low values (<0.1 W $m^{-1}$ $K^{-1}$) while preserving localized horizontal pathways, this porous architecture yields an exceptionally high AR despite modest absolute conductivity values.

It would be useful to determine a quantitative, empirical relationship between anisotropy ratio and the various processing, microstructural, and material parameters across different studies. However, establishing a single quantitative relationship across a wide range of composites remains challenging; existing literature exhibits wide variation in processing methods and sample form factors, often alongside incomplete filler characterization and reporting.

Overall, the analysis presented in this section highlights that both thermal conductivity and anisotropy in materials and composites are governed by a combination of intrinsic properties and microstructural design. Trends in thickness, loading fraction, and material class demonstrate that directional heat transport is highly sensitive to filler type, alignment, and matrix interactions. These observations underscore that optimizing thermal performance in composites requires deliberate control over microstructure rather than simply increasing filler content.

## 5 Electrical Properties of Dielectric Materials: Trends and Discussion

Beyond high thermal conductivity for heat dissipation for better thermal management in electronic and electrical systems, the materials must simultaneously possess good electrical insulation capabilities. The electrical properties that represent insulating capability include dielectric breakdown strength, dielectric constant, and volume resistivity. Supporting information compiles all the electrical data analyzed in this manuscript.

Dielectric strength ($E_{b}$) is defined as the maximum voltage, a material can endure before it transitions from an electrical insulator to a conductor. Evaluated under ASTM D149 guidelines [182], the testing apparatus typically sandwiches the sample between two electrodes submerged either in air or an insulating oil. The voltage is continuously increased until the material breaks down; this transition is marked by a sudden transformation from negligible leakage current to a sharp current spike, defining the breakdown voltage. Minimum acceptable breakdown strengths depend heavily on the operating voltage of the device, though a baseline of approximately 10 kV/mm is commonly targeted.

Dielectric constant ($\varepsilon^{\prime }$) is another property that represents the electrical insulation capability of the material. It is a dimensionless quantity that measures the material's capacity to store electrical energy through polarization when subjected to an external alternating electric field. For electrical insulation applications, a low dielectric constant is usually preferred to reduce stray capacitance and capacitive heating. To evaluate this property, ASTM D150 [183] outlines standard guidelines using a parallel‐plate capacitor configuration. During testing, a specimen is placed between two parallel plates, and an alternating voltage is applied; the dielectric constant is then determined by measuring the capacitance of the cell with the material present relative to its capacitance in a vacuum or air. This property is measured across a range of frequencies and generally exhibits a decreasing trend with the frequency. At lower frequencies, dipoles and space charges have sufficient relaxation time to align with the oscillating electric field. At high frequencies, these polarization mechanisms can no longer keep pace with the rapidly switching field, leading to a convergence of permittivity values. For standard electrical insulation and electronic substrate applications, a low dielectric constant (between 2 and 4) is typically targeted.

Volume resistivity serves as another critical indicator of a material's electrical insulation capability, representing its internal resistance to the flow of direct electrical current. Standard practices for measuring this are prescribed in ASTM D257 [184]. In this method, the testing material is placed between two electrodes, a potential difference is applied across its bulk, and the resulting current is recorded using an electrometer. Using the applied voltage (V), measured current (I), the sample's thickness (t), and the electrode's effective cross section area (A), the volume resistivity (ρ) is typically calculated as:

(2) $$\rho =\frac{A}{t}\frac{V}{I}.$$

For robust electrical insulation, volume resistivity values exceeding $10^{8}\;\Omega \cdot \text{cm}$ at their designated operating temperatures are typically targeted.

To provide a systematic overview of the current material landscape, Table 3 compiles these electrical properties for engineered materials based on meta‐aramid, polyester, cellulose, and polyimide, while Table 4 details the performance of various boron nitride (BN)‐based composites. An analysis of the dataset compiled in this study reveals that many existing works fail to consistently report all the three properties. In fact, numerous studies omit these critical parameters entirely. This inconsistency underscores the urgent need for both standardized electrical testing and comprehensive data reporting if these newly developed materials are to be effectively implemented in their intended applications.

**Table 3:** Summary of electrical properties of engineered materials based on meta‐aramid, polyester, cellulose, and polyimide. N.D. = no data reported.

			Measured electrical properties $E_{b}$ [kV/mm]			
Material	Material class	Thickness [μm]		$\varepsilon^{\prime }$	ρ [Ω·cm]	Refs.
Nomex 410	Meta‐aramid based	242	33	2.7	6 × 10 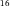	[42]
Nomex‐Mylar‐Nomex	Meta‐aramid/polyester	397	42.5	N.D.	N.D.	[185]
Fish paper	Cellulose based	397	11.8	N.D.	N.D.	[43]
3M^TM^ CeQUIN		250	7.2	N.D.	N.D.	[186]
Kapton HN	Polyimide based	76	205	3.5	1.40 × 10 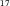	[187]
Kapton MT+		50	209	N.D.	1 × 10 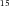	[188]
Kapton FN		50	197	2.7	2.3 × 10 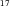	[189]
Kapton EN		50	375	N.D.	N.D.	[190]
Kapton MT		50	201	N.D.	1.00 × 10 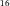	[191]
3M^TM^ TufQUIN	Polyester based	230	15	N.D.	N.D.	[185]
3M^TM^ ThermaVolt		250	20	N.D.	N.D.	[192]
Humidity resistant sheet		76	14.6	N.D.	N.D.	[193]

**Table 4:** Summary of electrical properties of engineered materials based on boron nitride nanofillers. N.D. = no data reported.

				Measured electrical properties			
Filler	Base matrix	Loading fraction [%]	Thickness [μm]	$E_{b}$ [kV/mm]	$\varepsilon^{\prime }$	ρ [Ω·cm]	Refs.
BNNS	PVDF	29.5	28	377	N.D.	3.87 × 10 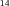	[71]
BNNS	PU	80	2000	N.D.	N.D.	5.10 × 10 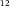	[11]
BNNS	PS	13.4	250	N.D.	N.D.	1.00 × 10 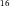	[57]
BNNS	Epoxy	15	2000	N.D.	N.D.	2.00 × 10 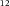	[72]
BNNS	PDMS	20	1000	34	N.D.	1 × 10 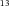	[172]
BNNS	PI	14.3	N.D.	N.D.	3.23	N.D.	[194]
BNNS	PMMA	69.4	300	N.D.	N.D.	3 × 10 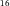	[145]
BNNS	PI	22	90	N.D.	3.49	N.D.	[144]
BNNS	Epoxy/PDA	24.2	2000	N.D.	N.D.	1 × 10 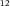	[81]
BNNS	PBO/PDA	30	30	356.67	4.39	N.D.	[61]
BNNS	TPU	36.4	85	184.5	N.D.	1.03 × 10 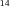	[56]
BNNS	PPGTD	24	75	N.D.	N.D.	1 × 10 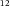	[55]
BNNS	PMMA/PN	40	N.D.	N.D.	N.D.	1 × 10 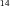	[138]
BNNT	PC	7.2	2000	N.D.	3.2	1 × 10 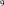	[62]
hBN	HDPE	23.2	3000	N.D.	3	1 × 10 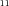	[80]
hBN	PDMS	40	100	N.D.	3.82	N.D.	[79]
hBN	PPSU	16	960	50	N.D.	N.D.	[173]
hBN	PVA	16	80	120	N.D.	7 × 10 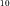	[77]
hBN	Epoxy	19.7	1000	N.D.	5.6	N.D.	[78]

Commercial benchmarking materials, including Nomex and the Kapton series of polyimide films exhibit exceptional electrical properties, as evidenced in Table 3. Widely utilized for slot liner applications in heavy‐duty electric motors, these industry standards are engineered to endure severe electromechanical stresses. Across all categories, these materials demonstrate excellent bulk insulation capabilities. They have volume resistivities well above $10^{15}\;\Omega \cdot \text{cm}$, low dielectric constants (below 4), and high dielectric breakdown strengths exceeding 10kV/mm. Notably, thin Kapton films can have $E_{b}$ as high as 200kV/mm, establishing a rigorous baseline for demanding industrial environments.

While these materials serve as excellent benchmarks for electrical insulation capabilities, these typically have low thermal conductivities, as discussed in Section 4. This highlights the need for next‐generation materials to exhibit good electrical as well as thermal properties. The reported electrical properties for the polymer composites containing BN fillers are listed in Table 4. Based on the compiled literature, these properties are governed by complex microstructural interactions.

The incorporation of boron nitride (BN) fillers into pristine polymer matrices has been widely demonstrated to maintain or explicitly enhance the breakdown strength ($E_{b}$) and the volume resistivity (ρ) due to the high intrinsic bandgap and excellent insulating characteristics of the BN fillers [195, 196]. As reported in [56, 71, 77], at optimal loading fractions, these high‐aspect‐ratio 2D fillers induce a distinct “scaffolding effect” within the matrix. The well‐dispersed BN networks act as mechanical and electrical barriers that severely impede charge carrier migration. This effectively delays electrical propagation and substantially improves $E_{b}$ and ρ. Enhancing filler dispersion within the polymer matrix [172] or optimizing interfacial binding between the phases [145] also serves as an effective strategy to minimize leakage current and increase electrical insulation.

A common trend observed across various composite systems is that these properties reach an upper limit near the percolation threshold. Past this point, they either stabilize or deteriorate, a phenomenon widely ascribed to the effects of filler agglomeration and the formation of interfacial voids due to additional loading [71, 172, 173]. Nevertheless, filler incorporation may also yield a negligible impact on the electrical insulation profile when both the pristine matrix and the reinforcement phase possess inherently high and comparable electrical resistivity [57, 80].

The dielectric constant is often dictated by the interfacial polarization of the matrix and filler phase. Maxwell‐Wagner‐Sillars Effect [197] states that when fillers possess a higher intrinsic dielectric constant than the host matrix (e.g., BNNTs or hBN relative to PC [78] or PDMS [80] or BNNS relative to PI [144]), the overall permittivity $\varepsilon^{\prime }$ of the composite inevitably increases. This is further reinforced by the high aspect ratio and substantial surface area of the nano‐fillers, which introduce a high density of phase interfaces, and therefore increases the dielectric loss. Conversely, introducing porosity helps in lowering the dielectric constant as air has $\varepsilon^{\prime }$ of 1.

Overall, the analysis established in this section underscores the intricate dependence of composite electrical insulation performance on the individual properties of the host matrix and filler phase, alongside deliberate microstructural design.

## 6 Summary and Perspectives

This review evaluates recent progress in developing electrically insulating thin films and sheets which exhibit enhanced thermal conductivity in the in‐plane or cross‐plane or both directions. The thermal properties of both bulk materials like BN, AlN, Si_3_N_4_, and Al_2_O_3_, which exhibit inherent thermal anisotropy, and engineered materials like polymers, fibers, and composites, which utilize a combination of intrinsic materials and appropriate fabrication methods and design to get the desired properties, are examined. Importantly, the work focuses on understanding the impact of different factors like matrix‐filler combination, volume fraction of fillers, thickness, and fabrication method to engineer the thermal conductivity and the anisotropy ratio of the engineered materials.

A clear hierarchy in thermal conductivity is observed across material systems: pure polymers occupy the low‐conductivity regime, intrinsic crystalline solids define the high end, and composites generally bridge the intermediate range depending on filler type, concentration, and alignment.

Special attention was devoted to BN‐based composites due to their unique combination of intrinsic anisotropy and tunable composite architectures, which make them particularly attractive for thermal management applications. Among these, polymer‐based BN composites dominate the literature, reflecting the widespread use and processability of polymer matrices in electronic packaging. The variation in polymer chemistry across systems significantly affects filler–matrix interfacial interactions, contributing to the broad spread in reported thermal conductivities and anisotropy ratios. Comparisons among BNNT, BNNS, and *h‐*BN‐based composites reveal that BNNT systems are the most challenging to fabricate, primarily due to their high aspect ratio, entangled morphology, and tendency toward agglomeration at higher loadings. This complex interplay between filler alignment, network connectivity, and matrix interactions illustrates that anisotropy is primarily governed by microstructural control rather than filler content alone.

In addition to the analysis of the trends in thermal properties, we also look at the observed trends in electrical properties like dielectric breakdown strength, dielectric constant, and volume resistivity. Like the thermal properties, even these electrical properties strongly depend on the microstructure design and the filler‐matrix interactions. The incorporation of high‐bandgap BN fillers generally maintains or enhances $E_{b}$ and ρ by acting as a structural scaffold that restricts charge carrier mobility, an effect optimized by homogeneous filler dispersion and robust interfacial binding. However, these insulating properties typically encounter a strict upper bound near the percolation threshold, beyond which filler agglomeration and interfacial voiding cause performance degradation. Furthermore, dielectric behavior is heavily governed by interfacial polarization via the Maxwell‐Wagner‐Sillars effect; the high surface area of high‐aspect‐ratio fillers inherently increases the composite dielectric constant ($\varepsilon^{\prime }$), though this can be effectively countered by introducing controlled sub‐micron porosity to lower the bulk dielectric constant.

### 6.1 A Strategic Framework for Co‐Designing Next‐Generation Insulating Materials

To bridge the gap between empirical literature trends and practical application, we propose a strategic framework for material co‐design. This framework translates the observed structure‐property relationships into concrete guidelines for co‐engineering both the thermal and electrical performance of next‐generation thin films and sheets.

1. **Thermal Management Design Framework**: Based on the analysis, a strategic framework to optimize directional heat dissipation and tailor the thermal anisotropy ratio involves:
  - **Anisotropy‐driven process selection and microstructure alignment**: Selecting scalable fabrication methodologies tailored directly to the target direction of heat dissipation. To maximize in‐plane thermal conductivity ($k_{∥}$) for lateral heat spreading, high‐shear processing techniques such as tape casting, solution casting, or hot‐pressing preceded by appropriate dispersion methods and electrospinning, must be utilized to forcibly align high‐aspect‐ratio fillers in the in‐plane direction. Conversely, to enhance cross‐plane dissipation ($k_{⊥}$) and circumvent the typical thermal bottlenecks of layered structures, field‐assisted assembly (magnetic or electric fields) or ice‐templating and freeze‐drying should be deployed to construct vertically oriented filler pathways.
  - **Interfacial engineering and dispersion optimization via surface functionalization**: Implementing targeted surface modification strategies to simultaneously mitigate the severe Kapitza resistance bottleneck and eliminate filler agglomeration at high volume fractions. Utilizing functionalized Boron Nitride architectures, such as chemically tailored Boron Nitride Nanocations (BNNCs) or silane‐coupled nanosheets, optimizes covalent/non‐covalent interfacial bonding with the host polymer. This enhances structural dispersion throughout the matrix, minimizes phonon‐scattering defects at the boundaries, and drastically reduces the interfacial thermal resistance that typically limits thermal conductivity scaling.
2. **Electrical Insulation Design Framework**: Based on the analysis, a strategic framework to enhance electrical insulation performance could involve:
  - **Controlled filler incorporation and dispersion**: Integrating Boron Nitride (BN) nanofillers and ensuring their homogeneous dispersion within the polymer matrix prevents localized electric field concentration and minimizes defects.
  - **Structural scaffold engineering**: Developing a well‐engineered filler network or tortuous scaffold effectively introduces deep charge‐trapping sites, impeding the mobility of charge carriers, and suppressing leakage currents under high electric fields.
  - **Hierarchical porosity design**: Strategically introducing controlled, sub‐micron porosity within the matrix can be leveraged to lower the bulk dielectric constant ($\varepsilon^{\prime }$) and mitigate capacitive heating, without sacrificing the material's structural or breakdown integrity.

### 6.2 Gaps in the Field

Despite substantial progress in achieving and reporting high thermal conductivities and tunable anisotropy, there remains a notable gap in the comprehensive characterization of these materials. As evidenced by the dataset generated in this study, the existing literature lacks standardized reporting of both in‐plane and cross‐plane thermal conductivities of the materials, their thicknesses, and the associated measurement uncertainties. In addition, many studies report impressive thermal performance yet overlook other important design characteristics. These include electrical performance (dielectric breakdown strength and dielectric constant), mechanical and environmental reliability (anisotropy under bending, thermal cycling, aging, and humidity), scalable manufacturing, and device‐level validation. A holistic evaluation is crucial to assess the true applicability of these materials in real‐world devices.

Moreover, relatively few studies have explored in‐plane anisotropy, an increasingly relevant consideration for next‐generation devices that demand directional heat spreading within the plane. Techniques such as fiber alignment or directional filler orientation present promising pathways to engineer in‐plane anisotropy and should be pursued in future investigations.

In summary, the field has made substantial strides in the design and understanding of anisotropic thermally conductive materials. Continued progress will rely on the integration of advanced fabrication techniques, standardized measurement protocols, and comprehensive multi‐property characterization to enable the rational design of next‐generation electrically insulating materials with finely tuned anisotropic thermal behavior for diverse thermal management applications.

## Conflicts of Interest

The authors declare no conflicts of interest.

## Supporting information




**Supporting File**: smtd71012‐sup‐0001‐SuppMat.xlsx.

## Data Availability

Data supporting the findings of this study are available from the corresponding author on a reasonable request.
